# Putative regulation mechanism for the *MSTN* gene by a CpG island generated by the SINE marker Ins227bp

**DOI:** 10.1186/s12917-015-0428-3

**Published:** 2015-06-23

**Authors:** René van den Hoven, Emre Gür, Manuela Schlamanig, Martin Hofer, Ali Cesur Onmaz, Ralf Steinborn

**Affiliations:** Department of Companion Animals and Horses, University Equine Hospital, Vetmeduni Vienna, Vienna, Austria; Jockey Club of Turkey, Istanbul, Turkey; Genomics Core Facility, VetCore, Vetmeduni Vienna, Vienna, Austria; Department of Internal Medicine, University of Erciyes, Kayseri, Turkey

## Abstract

**Background:**

A single nucleotide polymorphism (SNP) in the first intron of the myostatin gene (*MSTN*) is associated with aptness of elite Thoroughbreds to race over sprint, middle or long distances. This intronic marker (g.66493737 T ≻ C), a short interspersed nuclear element (SINE) of 227 bp (Ins227bp) insertion polymorphism in the *MSTN* promoter, and the adjacent SNP BIEC2-417495 have not been studied for their association with racing aptness of the average Thoroughbreds raced in countries with lower status of the racing industry. This study investigated these markers regarding their prevalence and association with performance in common race horses. Markers were genotyped by amplification refractory mutation system-quantitative PCR (ARMS-qPCR) or amplicon melting. Furthermore, we asked whether the Ins227bp marker might theoretically regulate the expression of myostatin by generating a novel target for DNA methylation or by changing binding sites for transcription factors. Putative sites for DNA methylation or binding of transcription factors were predicted by MethPrimer and by the softwares JASPAR, MatInspector and UniPROBE, respectively.

**Results:**

Pairwise linkage disequilibrium between g.66493737 T ≻ C and Ins227bp was high (*r*^*2*^ = 0.93). A lower linkage was determined for g.66493737 T ≻ C and BIEC2-417495 (*r*^*2*^ = 0.69) as well as for BIEC2-417495 and Ins227bp (*r*^*2*^ = 0.76). The estimated frequencies for the presence of Ins227bp (I) indel and the C alleles at g.66493737 T ≻ C and BIEC2-417495 were 0.46, 0.47 and 0.43, respectively. Heterozygotes represented the most abundant genotype at each locus. The best racing distance (BRD) was significantly different between the homozygotes of each SNP (*p* = 0.01 to 0.03). C allele homozygotes at BIEC2-417495 or g.66493737 T ≻ C, as well as Ins227bp homozygotes earned most money on a mean distance ranging from 1211 to 1230 m. Heterozygotes earned most money on races over 1690 to 1709 m. The BRD for the T/T carriers at both SNP loci and for the SINE-free genotype was 1812 to 1854 m. Other performance parameters were not significantly different between the genotypes, except of the relative success score (RSS). The RSS was significantly slightly better on a distance of ≤1300 m for all carriers of the C allele and the Ins227bp compared to homozygous T genotypes and SINE-negative horses (*p* = 0.037 to 0.046). For distances of more than 1300 m the RSS was not significantly different between genotypes.

*In silico* assessment indicated that the Ins227bp promoter insertion might have generated a CpG island and a few novel putative binding sites for transcription factors.

**Conclusions:**

All three target polymorphisms (Ins227bp, g.66493737 T ≻ C, BIEC2-417495) are suitable markers to assess the ability of non-elite Thoroughbreds to race at short or longer distances. The CpG island generated by Ins227bp may cause training-induced silencing of *MSTN* expression.

**Electronic supplementary material:**

The online version of this article (doi:10.1186/s12917-015-0428-3) contains supplementary material, which is available to authorized users.

## Background

The g.66493737 T ≻ C marker located in the first intron of the *MSTN* gene predicts the racing ability of Thoroughbreds based on the quantitative traits best racing distance (BRD) or win-race distance [1–3]. C/C homozygotes appear better suited for fast, short-distance races (≤1300 m), whereas C/T genotypes seem to compete better in middle-distance races (1301 to 1900 m), while T/T homozygotes perform generally better over longer distances (>2114 m) [1, 2]. For two cohorts of elite horses a strong association was demonstrated for the C and T alleles with the sprinting or staying performance, respectively [3, 4].

**Fig. 1:**
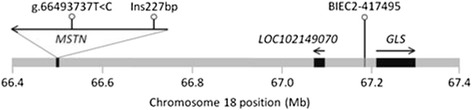
Location of the target polymorphisms on chromosome 18. Only the genes (in italics) most closely surrounding the three markers are depicted

A distant SNP, BIEC2-417495 (Fig. 1), located 692 kb or 30 kb upstream of *MSTN* or glutaminase (*GLS*) genes, respectively, is similarly associated with racing aptness [5].

The highest standard and most valuable elite Flat races are known as Group (Stakes) races, whereas Listed races are the next in status. The elite Thoroughbreds described before had won at least one Group race or a Listed race. Most previous studies have been performed with elite cohorts from countries with the most internationally regarded Thoroughbred industry. Such cohorts likely do not represent the population of Thoroughbreds raced in countries in which horse racing is regarded to be of poorer quality on an international level. It would be very interesting to see if the association between *MSTN* markers and best racing distance or other performance indicators holds true in a less well regarded Thoroughbred population. Therefore, we present an observational study on the previously identified variants in the equine *MSTN*, thought to influence the racing ability of Thoroughbred horses. For this we studied a cohort of 56 non-elite Thoroughbreds raced in Austria and Turkey. Races run were usually handicap races or other non-Group or non-Listed races.

It is currently not understood how the g.66493737 T ≻ C polymorphism, located in the middle of a relatively large intron (1.829 bp), may influence the expression of genes involved in the development of juvenile and mature equine muscles. Moreover, although some marginal increase in muscle mass has been described [6], the massive increase in muscle mass seen in other species with *MSTN* missense or nonsense mutations such as in knock-out mice [7], double muscled cattle [8, 9] or “bully” whippets [10] was not observed. The SINE of the *MSTN* promoter, Ins227bp, is in high linkage disequilibrium (*r*^*2*^ = 0.73 to 1) with the C allele at g.66493737 T ≻ C [2, 11], but considered less appropriate to predict racing aptness [2]. Recently, haplotype data suggested that Ins227bp is contemporary to and arose upon a haplotype containing the C allele at g.66493737 T ≻ C [11]. Moreover, it is suggested that Ins227bp, rather than the intron 1 SNP of *MSTN*, drives muscle fiber type characteristics and is the variant targeted by selection for short-distance racing [11].

To find a possible mechanism for this, we analysed the sequence *in silico* to identify putative binding sites for DNA methylation and transcription factors resulting from insertion of the Ins227bp polymorphism.

## Results and Discussion

### Linkage disequilibrium and allelic distribution

Compared to the study by Hill *et al.* [2] our experimental cohort of average Thoroughbreds was different in linkage disequilibrium pairwise tested for g.66493737 T ≻ C and Ins227bp as well as for g.66493737 T ≻ C and BIEC2-417495 (*r*^*2*^ values of 0.73 *versus* 0.93 and 0.86 *versus* 0.69, respectively see Additional file 1: Figure S1). The lower disequilibrium observed between g.66493737 T ≻ C and BIEC2-417495 makes it less difficult to assess the functional impact of either locus independent of the other. Table 1 displays the distribution of the Ins227bp, g.66493737 T ≻ C and the BIEC2-417495 alleles in the cohort of 56 non-elite Thoroughbreds.

**Table 1:** Distribution of marker alleles across the cohort of non-elite horses (n = 56)

BIEC2-417495	Ins227bp	g.66493737C/T
18 T/T	14 N/N 4 I/N	13 T/T 5 C/T
28 C/T	27 I/N	26 C/T
	1 II	2 C/C
10 C/C	8 I/I 2 IN	8 C/C 2 C/T

The estimated frequencies for the presence of Ins227bp (I) indel and the C alleles at g.66493737 T ≻ C and BIEC2-417495 were 0.46, 0.47 and 0.43, respectively. Heterozygotes represented the most abundant genotype for all mutations (Ins227bp: 59 % I/N, 16 % I/I and 25 % N/N; g.66493737 T ≻ C: 59 % C/T, 18 % C/C and 23 % T/T; BIEC2-417495: 50 % C/T, 18 % C/C and 32 % T/T).

### Performance indicators

There was no statistically significant difference in victories, places and shows, starts, life earnings, best earnings in a race and average earning per start between the genotypes for each marker (Table 2). However, the BRD was significantly different between some of the genotypes (Table 2). The RSS was calculated for distances of ≤ 1300 m (short) and > 1300 m (Table 3). On the short distance, the RSS determined for the C/C and C/T genotypes at g.66493737 T ≻ C was significantly higher compared to T/T carriers (*p* = 0.037 and *p* = 0.046, respectively). I/I genotypes had a marginally significant better RSS than the N/N genotypes (*p* = 0.052). For the BIEC2-417495 genotypes no difference in RSS was found for the short distance neither for distances more than 1300 m.

**Table 2:** Mean ± sd of the performance indicators per marker genotype

	BIEC2-417495	INS227bp	g.66493737 T ≻ C
	**C/C**	**I/I**	**C/C**
Victories	2.2 ± 2.5	2.1 ± 2.5	1.9 ± 2.4
Place & shows	8.6 ± 7.7	9.1 ± 7.7	8.3 ± 7.7
Races run	20.7 ± 14.9	18.8 ± 11.7	17.6 ± 11.77
Life earnings (€)	20261 ± 18749	22860 ± 18398	20638 ± 18714
**Best racing distance (m)**	**1211 ± 537** ^**a**^	**1211 ± 537** ^**b,c**^	**1230 ± 510** ^**d,e**^
Best race earnings (€)	5578 ± 5708	5992 ± 5797	5457 ± 5722
Earnings/start (€)	919 ± 917	1,214 ± 961	1102 ± 973
	**C/T**	**I/N**	**C/T**
Victories	2.3 ± 2.3	2.6 ± 2.9	2.7 ± 2.9
Place & shows	6.6 ± 6.1	6.7 ± 5.8	6.8 ± 5.8
Races run	17.5 ± 12.5	19.8 ± 18.7	19.8 ± 18.7
Life earnings (€)	28784 ± 32105	28,823 ± 33785	29226 ± 33513
**Best racing distance (m)**	1690 ± 361	**1709 ± 347** ^**b**^	**1705 ± 351** ^**d**^
Best race earnings (€)	7890 ± 8184	7376 ± 7818	7663 ± 7730
Earnings/start (€)	1626 ± 1909	1441 ± 1812	1498 ± 1797
	**T/T**	**N/N**	**T/T**
Victories	3.1 ± 4.0	2.7 ± 3.4	2.9 ± 3.5
Place & shows	8.4 ± 10.1	8.4 ± 10.4	8.9 ± 10.7
Races run	22.4 ± 25.0	19.8 ± 19.0	20.8 ± 19.4
Life earnings (€)	30382 ± 43537	28471 ± 43456	29620 ± 45009
**Best racing distance (m)**	**1822 ± 416** ^**a**^	**1812 ± 461** ^**c**^	**1854 ± 454** ^**e**^
Best race earnings (€)	6074 ± 6655	6297 ± 7127	6028 ± 7343
Earnings/start (€)	1122 ± 1272	1162 ± 1374	1103 ± 1412

^a^p = 0.01

^b^p = 0.03 ^c^p = 0.02

^d^p = 0.03 ^e^p = 0.01

Corresponding letters per column indicate significance pairwise difference (Dunn’s Test)

**Table 3:** Mean and standard deviation of RSS per genotype for sprint and longer distance and number of starts on these distances

	BIEC2-417495	INS227bp	g.66493737 T ≻ C
	**C/C**	**I/I**	**C/C**
RSS for:			
≤1300 m	3.5 ± 2.3	**3.6 ± 2.5** ^**a**^	**3.3 ± 2.5** ^**b**^
>1300 m	2.6 ± 1.6	2.8 ± 3.0	2.6 ± 1.6
races ≤1300 m	8.6 ± 10.0	8.5 ± 10.7	7.8 ± 10.4
races >1300 m	10.2 ± 9.4	8.8 ± 5.5	8.3 ± 5.4
	**C/T**	**I/N**	**C/T**
RSS for:			
≤1300 m	2.6 ± 2.9	2.8 ± 3.0	**3.0 ± 3.0** ^**c**^
>1300 m	2.9 ± 1.4	2.8 ± 3.0	2.8 ± 1.4
races ≤1300 m	2.8 ± 3.4	2.9 ± 3.2	3.0 ± 3.2
races >1300 m	14.2 ± 10.9	16.3 ± 17.5	16.3 ± 17.5
	**T/T**	**N/N**	**T/T**
RSS for:			
≤ 1300 m	2.0 ± 2.8	**1.4 ± 1.9** ^**a**^	**1.1 ± 1.7** ^**b,c**^
>1300 m	2.8 ± 1.3	2.9 ± 1.3	3.0 ± 1.3
races ≤ 1300 m	3.2 ± 6.0	4.0 ± 6.1	3.5 ± 7.0
races >1300 m	19.7 ± 23.0	15.3 ± 15.8	18.0 ± 16.1

^a^ *p* = 0.05

^b^ *p* = 0.037 ^c^ *p* = 0.046

Corresponding letters within a column indicate significance pairwise difference (Dunn’s Test)

Sampling bias in this study could not be prevented since assessment of the racing ability was based on results of races run on different tracks under different circumstances and over a wide range of distances. This forced us to cluster race distance slightly differently as was done by others [1, 3]. Considering maximum speed of a Thoroughbred, a real sprint distance should not be more than 1000 m [12]. We chose 1300 m as the nearest suitable approximation of a sprint distance to obtain a sufficient number of performances data. The same reason requested others to make a slightly different split at 1600 m [1]. Existing data provide evidence that the proportion of anaerobic power decreases to less than 5 % if races are 2400 m or longer [13]. Thus, the empirical classification of distances ranging between 1000 and 2400 m according to the International Federation of Horseracing Authorities (www.horseracingintfed.com) should be regarded as arbitrary. In this respect, the BRD for the C/C (and I/I) genotypes on average fell within the physiological “sprint” distance (<1400 m). Ranges of BRD between the C/T (I/N) and T/T (N/N) genotypes did overlap considerably, as was also reported by others [1]. This is plausible since in addition to genotype many more factors determine the racing success of a horse. Nevertheless, the pattern confirms the underlying genetic aptness for a specific distance and could be used by the trainer to strategically design a horse’s racing career.

Horses were identified as non-elite due to their non-competing status in Group or Listed races. However, there was a large variation in price money won and some might have become elite horses in the hands of other trainers. We tried to estimate the strength of the associations of the genotypes and racing aptness in the general horse population, however the sample size of 56 horses was too small to allow further analyses of association between genotype and racing performance. Sample sizes of at least 200 horses and even more than 4500 in case of victories would have been needed to obtain a minimal power of 0.80. Therefore, it is not surprizing that in other studies with larger cohorts BRD was often the only trait that was significantly associated with genotype [1–4]. Although our BRD was not based on winning races, instead being determined by distance of race in which the horse earned most money, the association with the genotypes of g.66493737 T ≻ C in our non-elite race horse population agrees with that described for cohorts of elite and better quality horses [1–4]. The proportion of C/C homozygotes in our non-elite cohort was dissimilar to those given by Hill et al. [2] (18 % *versus* 29 %), but similar to that of Tozaki et al. [4]. The proportion of T/T homozygotes in our cohort was similar to that of Hill et al. [2] but smaller than that of Tozaki et al. [4] (23 % *versus* 31 %), likely explained by the different origins of the populations.

The Nearctic-Northern Dancer sire line is strongly associated with dispersion of the C/C genotype at g.66493737 T ≻ C [11]. Our cohort did not confirm this finding. The mean percentage of Nearctic blood in our g.66493737 T ≻ C C/C horses was not higher (*p* = 0.4) than in the C/T and T/T horses. Similar trends were found for the other two markers (data not shown).

The C allele is not unique for Thoroughbreds and Thoroughbred-derived populations. It was even found at a high frequency in Shetland ponies (0.32 to 0.50) and Fulani horses (0.33) [11, 14]. In contrast, the Ins227bp marker appears to be more specific for Thoroughbreds, Quarter horses and related breeds and is distributed across other breeds only at minor frequency [11, 15].

The reason of the statistical association of the *MSTN* polymorphism with racing aptness is still unknown because the strongest marker for this trait, BIEC2-417495 [2], is located far upstream (692 kb) of *MSTN* near the locus of the glutaminase (*GLS*) gene. This mitochondrial enzyme is assumed to play a role in energy production. So far, this gene or its alleles have not been studied in the horse (www.omin.org/entry/138280).

Nevertheless, the C allele of g.66493737 T ≻ C is regarded as a marker for muscularity [1–4]. Inconsistently, the tightly associated Ins227bp insertion polymorphism [2, 11], however, was not found to affect muscle mass [16]. Thus, a possible effect of the C allele on muscle mass needs further confirmation. Although the *MSTN* polymorphisms may not clearly affect mature muscle mass, they might influence prenatal muscle differentiation and juvenile composition. In Quarter Horses and Thoroughbreds the C allele at g.66493737 T ≻ C as well as the Ins227bp marker appear to be associated with higher and lower proportions of type 2X and type I fibres, respectively [11, 15]. Thus, Ins227bp could indicate the potential for high speed of Thoroughbreds too. Interestingly, Thoroughbreds being homozygous for the C allele at g.66493737 T ≻ C showed rather a higher transcript expression of *MSTN* in a non-trained condition compare to the C/T and T/T type. Only after a period of 10 months of training the expression level decreased to similar levels as the C/T and T/T genotypes [17]. This contradicts the simplistic hypothesis that a decreased *MSTN* expression leads to increased muscle mass. Theoretically, the three target polymorphisms could cause a change of *MSTN* expression by intron mediated enhancement [18–20], a distant regulatory DNA element located several hundred kilobases away [21], or by a genetic or epigenetic change of the *MSTN* promoter.

### Novel transcription factor binding site candidates and CpG island caused by Ins227bp

**Fig. 2:**
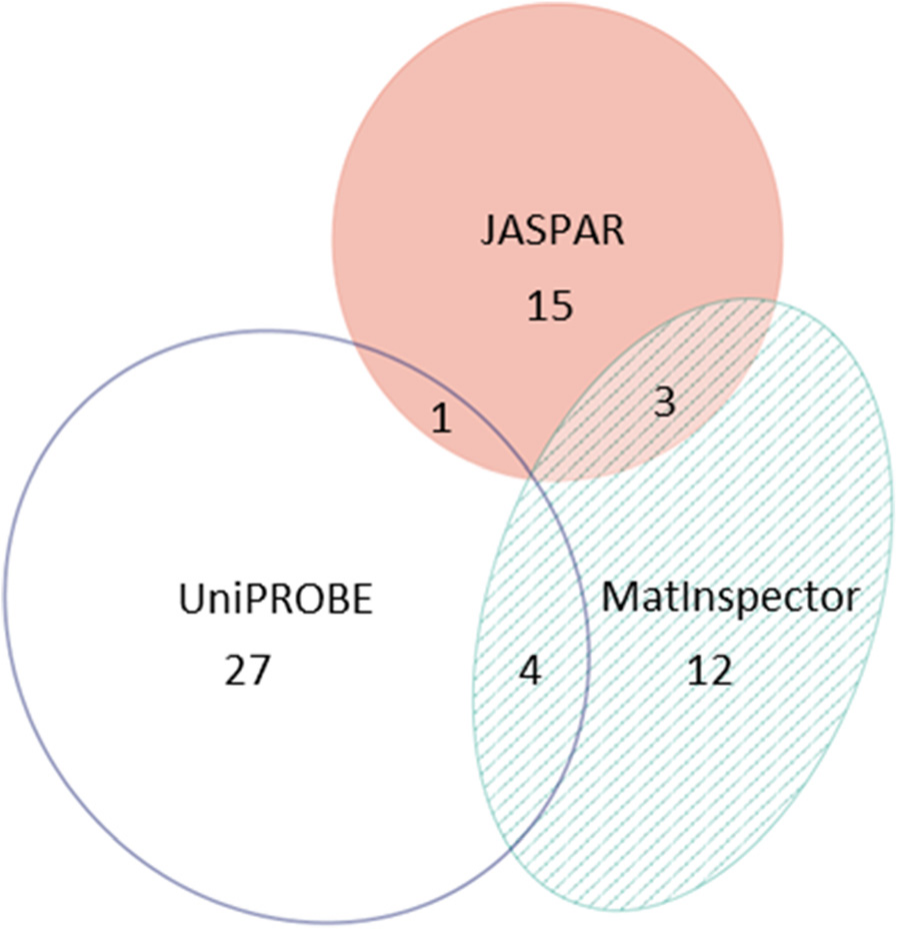
Insertion of the SINE marker Ins227bp into the equine *MSTN* promoter created one, three or four putative transcription factor binding sites according to the pairwise intersections of predictions obtained by the software tools JASPAR, MatInspector and UniPROBE. The pairwise intersections contained Nkx3-2 and the closely related Nkx3-1 (JASPAR crossed with UniPROBE), Nkx2-5, ZNF354 and MZF1 (JASPAR with MatInspector), as well as PlagI1 (twice), ZNF300 or nearly identical ZNFs and Nkx2-5 (MatInspector with UniPROBE). The Venn diagram was generated with eulerAPE 3.0.0 [ Micallef L, Rodgers P: eulerAPE: Drawing Area-Proportional 3-Venn Diagrams Using Ellipses. *PLoS ONE 2014*, 9: e101717]

**Fig. 3:**
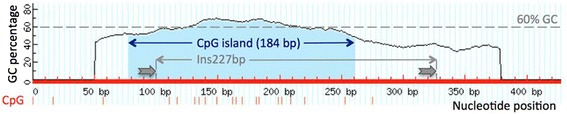
Inserting the Ins227bp SINE into the *MSTN* promoter generates a novel CpG island. The 184 bp island (nucleotides 78 to 261 highlighted by light blue background) was identified by the MethPrimer software. Red bars designate CpG dinucleotides. The integration-flanking set of 15 bp direct repeats, TAAAAAGCCACTTGG, one being part of and the other being adjacent to the SINE insertion, is depicted by arrows

It was not very surprising that the insertion of the 227 bp SINE (Ins227bp) into the promoter of the *MSTN* gene generated some novel putative binding sites for transcription factors . In more detail, whereas the insertion did not erase a putative transcription factor binding site according to the analysis tools JASPAR, MatInspector and UniPROBE applied under stringent settings, it created one, three or four novel putative transcription factor binding sites according to the pairwise intersections of the three prediction programs (Fig. 2). There was no site predicted by all three tools. More surprising, however, was the finding that the Ins227bp insertion created a novel CpG island (Fig. 3) including a downstream segment at the insertion site.

Gene expression differences that are the result of SINE insertions are likely to be a recurrent theme in the study of complex traits [22], however, so far very few studies have conclusively demonstrated exaptation of transposable elements as transcriptional regulatory regions [23]. Their functioning as nucleation centres for de novo methylation is striking in an epigenetic context [24]. Further dissecting the effects of the genetic variants will benefit understanding the regulation of the racing ability of Thoroughbreds. Of special interest in this regard would be, to unravel whether the SINE Ins227bp of the *MSTN* promoter would regulate *MSTN* expression via the generated CpG island and/or via changed target sites for transcriptional regulator(s).

## Conclusion

Each of the the three polymorphisms studied represents a suitable genetic marker to predict the sprinting ability of non-elite Thoroughbreds. Future experiments with large numbers of horses, between 200 to over 4500, depending on the studied trait should address the possible role of the SINE insertion Ins227bp as a putative *cis* element enabling transcriptional regulation via association with trans-acting factors and/or modulation by exercise. The use of untrained age-matched controls will exclude that methylation regulates expression of *MSTN* in an age-dependent manner in horses of 20 and 30 months [17].

## Methods

### Animals and samples

Roots from hair samples were collected from Thoroughbreds in Austria (*n* = 20) and Turkey (*n* = 36). The life time performance of these horses was extracted from published race results.

### Genotyping assays

The SNPs g.66493737 T ≻ C and BIEC2-417495 were typed by ARMS-qPCR) [25]. The length polymorphism Ins227bp was analysed by amplicon dissociation and agarose gel electrophoresis.

Primers (Additional file 2: Table S1) were designed with the software Primer Express 2.0 (Life Technologies, Foster City, USA) and controlled for dimer formation using the web tool NetPrimer (www.premierbiosoft.com/netprimer/). Their specificity was evaluated with Primer-BLAST of NCBI using the “nr“ database of *Equus caballus*. The secondary structure of the PCR product was analysed with the Mfold software [26].

Genomic DNA was extracted from hair roots using the NucleoSpin® Tissue Kit according to the manufacturer’s instructions (Macherey-Nagel GmbH & Co. KG, Düren, Germany). DNA concentration was measured spectrophotometrically using the Hellma® TrayCell (Hellma Analytics, Müllheim, Germany) on the BioPhotometer 6131 (Eppendorf, Hamburg, Germany). Sample concentrations ranged between 2 and 11 ng/μl. Amplification was performed in duplicate 20-μl reactions. A single reaction consisted of 1 × reaction buffer (70 mM Tris–HCl (pH 8.3), 50 mM KCl, 10 mM (NH_4_)_2_SO_4_, 0.1 mg/ml gelatin), 3 mM MgCl_2_, 0.2 mM of each dNTP, 200 nM of each primer (Solis Biodyne, Tartu, Estonia), 1 unit hot-start *Taq* DNA polymerase (HOT FIREPol® DNA Polymerase; Solis Biodyne, Tartu, Estonia), 3 μl DNA and 0.4 × EvaGreen (Biotium, Hayward, USA) or 200 nM hydrolysis probe depending on the detection format used (Additional file 2: Table S1). Cycling conditions on the StepOnePlus^™^ Real-Time PCR System (Life Technologies) running under the software version 2.0 were 95 °C for 15 min followed by 45 cycles of 95 °C for 15 s, 58 °C for 20 s, and 60 °C for 30 s. For dye-based qPCR (markers: Ins227bp and g.66493737 T ≻ C) amplicon dissociation analysis from 60 °C to 95 °C with 0.3 °C/s increments and continuous acquisition of fluorescence was performed. Specific amplification was concluded when the target and the no-template control showed different melting temperatures. In addition, the amplicon of the Ins227bp assay was assessed on an 1 % agarose gel stained with a 10.000-fold dilution of the dye Midori Green Advance (Biozym Scientific GmbH, Hessisch Oldendorf, Germany) and visualised on the AlphaImager HP System (Biozym Scientific GmbH, Hessisch Oldendorf, Germany) equipped with a blue light screen.

A sample was considered homozygous or heterozygous if the difference of the quantification cycle (*Cq*) values obtained by the two discriminative assays of ARMS-qPCR was ≥ 7 or ≤ 2.5, respectively.

### Pairwise testing of linkage disequilibrium

Haploview 4.2 was used for pairwise testing of linkage disequilibrium [27].

### Prediction of transcription factor binding sites putatively created by the Ins227bp insertion

Transcription factor binding sites putatively created by the SINE insertion Ins227bp were analysed by the software tools JASPAR (version 5.0_ALPHA) [28, 29], MatInspector (version 8.2) [30] and UniPROBE (state of March 2015; [31] calling upon different databases. To report only the most likely sites stringent thresholds were applied, namely a 90 % relative profile score threshold for JASPAR set to “CORE Vertebrata”, a core similarity of 1.0 and a matrix similarity of at least 0.95 for MatInspector when set to vertebrates and a score threshold of 0.48 for UniPROBE set to mammalian which is slightly below the maximum value of 0.50.

### CpG island prediction

The CpG island was predicted by the MethPrimer software [32] using an island size of at least 100 nucleotides, a GC percentage of at least 50 % and an observation/expectation CpG ratio of more than 0.6.

### Calculation of relative success scores (RSS)

The various racing distances on which the horses had performed could only suitably be clustered into: sprint distance (≤1300 m) and non-sprint (>1300 m). A RSS was calculated for each distance class. The algorithm for the RSS was to sum up all points obtained in the respective distance class, divided by the number of starts in that class. Wins were given ten points, a 2nd place five, a 3rd place four, a 4th place three, a 5th place two and unplaced start was given one point. In this scoring system wins are twice as important as a second place, while honouring a finished race with one point allowed to include the effects of frequent starts and indicates a certain level of toughness. Furthermore, per genotype group the mean victories, mean places and shows, mean number of starts, mean life earnings, mean best racing distance based on highest earnings, mean best earnings in a race and mean earnings per start were calculated. The percentage of Nearctic blood in the pedigree (*F*_*x*_) was calculated by the term Σ [0.5]^*x1*+*x2*+1^ [33] whereby *x1* represents the number of generations from sire(s) to Nearctic and *x2* the number of generations from dam(s) to Nearctic. The parameters were used to identify possible associations between Ins227bp and genotypes at BIEC2-417495 and g.66493737 T ≻ C.

### Statistics

Statistical analysis was performed using IBM® SPSS® version 20 (IBM Corporation, New York, United States) statistical software. All data were tested by Shapiro-Wilks test and appeared not normally distributed (*p* < 0.04). Parameter differences between the genotypes at each of the three markers were analysed by a Kruskall-Wallis H omnibus tests and significant results (*p* < 0.05) were further subjected to post hoc rank tested using the Dunn’s pairwise test with Bonferroni adjustment for multiple comparisons.

### Ethics statement

All animal procedures were approved by the Animal Research Ethics Committee of the University of Veterinary Medicine Vienna (Austria). Hair samples were collected with informed consent of the owner or with trainer’s consent acting on behalf of the owner.

## Additional files

Additional file 1: Figure S1. Llinkage disequilibrium pairwise tested for g.66493737 T ≻ C and Ins227bp as well as for g.66493737 T ≻ C and BIEC2-417495. Additional file 2: Table S1. Details of genotyping assays.

## References

[1] Hill EW, Gu J, Eivers SS, Fonseca RG, McGivney BA, Govindarajan P (2010). A sequence polymorphism in MSTN predicts sprinting ability and racing stamina in thoroughbred horses. PLoS One.

[2] Hill EW, McGivney BA, Gu J, Whiston R, MacHugh DE (2010). A genome-wide SNP-association study confirms a sequence variant (g.66493737C>T) in the equine myostatin (MSTN) gene as the most powerful predictor of optimum racing distance for Thoroughbred racehorses. BMC Genomics.

[3] Tozaki T, Miyake T, Kakoi H, Gawahara H, Sugita S, Hasegawa T (2010). A genome-wide association study for racing performances in Thoroughbreds clarifies a candidate region near the MSTN gene. Anim Genet.

[4] Tozaki T, Hill EW, Hirota K, Kakoi H, Gawahara H, Miyake T (2012). A cohort study of racing performance in Japanese Thoroughbred racehorses using genome information on ECA18. Anim Genet.

[5] Binns MM, Boehler DA, Lambert DH (2010). Identification of the myostatin locus (MSTN) as having a major effect on optimum racing distance in the Thoroughbred horse in the USA. Anim Genet.

[6] Tozaki T, Sato F, Kurosawa M, Hill EW, Miyake T, Endo Y (2011). Sequence variants at the myostatin gene locus influence the body composition of Thoroughbred horses. J Vet Med Sci.

[7] McPherron AC, Lawler AM, Lee SJ (1997). Regulation of skeletal muscle mass in mice by a new TGF-beta superfamily member. Nature.

[8] McPherron AC, Lee SJ (1997). Double muscling in cattle due to mutations in the myostatin gene. Proc Natl Acad Sci U S A.

[9] Grobet L, Poncelet D, Martin LJ, Royo LJR, Brouwers B, Pirottin D (1998). Molecular definition of an allelic series of mutations disrupting the *myostatin* function and causing double-muscling in cattle. Mamm Genome.

[10] Mosher DS, Quignon P, Bustamante CD, Sutter NB, Mellersh CS, Parker HG (2007). A mutation in the myostatin gene increases muscle mass and enhances racing performance in heterozygote dogs. PLoS Genet.

[11] Petersen JL, Stephanie J, Valberg SJ, Mickelson JR, McCue ME (2014). Haplotype diversity in the equine myostatin gene with focus on variants associated with race distance propensity and muscle fiber type proportions. Anim Genet.

[12] Nielsen BD, Turner KK, Ventura BA, Woodward AD, O‘Connor CI (2006). Racing speeds of quarter horses, thoroughbreds and Arabians. Equine Vet J Suppl.

[13] McMiken DF (1983). An energetic basis of equine performance. Equine Vet J.

[14] Bower MA, McGivney BA, Campana MG, Gu J, Andersson LS, Barrett E (2012). The genetic origin and history of speed in the Thoroughbred racehorse. Nat Commun.

[15] Petersen JL, Mickelson JR, Rendahl AK, Valberg SJ, Andersson LS, Axelsson J (2013). Genome-Wide Analysis Reveals Selection for Important Traits in Domestic Horse Breeds. PLoS Genet.

[16] SanGiacomo NE: The Impact of Myostatin Genetic Polymorphism on Muscle Conformation in the Horse. PhD Thesis, Cornell University, College of Agriculture and Life Sciences, Animal Science; 2013.

[17] McGivney BA, Browne JA, Fonseca RG, Katz LM, MacHugh DE, Whiston R (2012). MSTN genotypes in Thoroughbred horses influence skeletal muscle gene expression and racetrack performance. Anim Genet.

[18] Bianchi M, Crinelli R, Giacomini E, Carloni E, Radici L, Yin Y (2013). Intronic Binding Sequences and Splicing Elicit Intron-Mediated Enhancement of Ubiquitin C Gene Expression. PLoS One.

[19] Parra G, Bradnam K, Rose AB, Korf I (2011). Comparative and functional analysis of intron-mediated enhancement signals reveals conserved features among plants. Nucleic Acids Res.

[20] Park SG, Hannenhali S, Chai SS (2014). Conservation in first introns is positively associated with the number of exons within genes and the presences of regulatory epigenetic signal. BCM Genomics.

[21] Guenther CA, Tasic B, Luo L, Bedell MA, Kingsley DM (2014). A molecular basis for classic blond hair color in Europeans. Nat Genet.

[22] Palmer AA, Dulawa SC (2010). Murine warriors or worriers: the saga of Comt1, B2 SINE elements, and the future of translational genetics. Front Neurosci.

[23] de Souza FS, Franchini LF, Rubinstein M (2013). Exaptation of transposable elements into novel cis-regulatory elements: is the evidence always strong?. Mol Biol Evol.

[24] Arnaud P, Goubely C, Pelissier T, Deragon JM (2000). SINE retroposons can be used in vivo as nucleation centers for de novo methylation. Mol Cell Biol.

[25] Steinborn R, Schinogl P, Zakhartchenko V, Achmann R, Schernthaner W, Stojkovic M (2000). Mitochondrial DNA heteroplasmy in cloned cattle produced by fetal and adult cell cloning. Nat Gene.

[26] Zuker M (2003). Mfold web server for nucleic acid folding and hybridization prediction. Nucleic Acids Res.

[27] Barrett JC, Fry B, Maller J, Daly MJ (2005). Haploview: analysis and visualization of LD and haplotype maps. Bioinformatics.

[28] Sandelin A, Alkema W, Engström P, Wasserman WW, Lenhard B (2004). JASPAR: an open-access database for eukaryotic transcription factor binding profiles. Nucleic Acids Res.

[29] Mathelier A, Zhao X, Zhang AW, Parcy F, Worsley-Hunt R, Arenillas DJ, Buchman S, Chen CY, Chou A, Ienasescu H, Lim J, Shyr C, Tan G, Zhou M, Lenhard B, Sandelin A, Wasserman WW. JASPAR 2014: an extensively expanded and updated open-access database of transcription factor binding profiles. Nucleic Acids Research 2014,42: D142-D147. 10.1093/nar/gkt997 PMC3965086 24194598

[30] Quandt K, Frech K, Karas H, Wingender E, Werner T, MatInd and MatInspector (1995). New fast and versatile tools for detection of consensus matches in nucleotide sequence data. Nucleic Acids Res.

[31] Hume MA, Barrera LA, Gisselbrecht SS, Bulyk ML, UniPROBE (2015). Update 2015: new tools and content for the online database of protein-binding microarray data on protein-DNA interactions. Nucleic Acids Res.

[32] Li LC, Dahiya R (2002). MethPrimer: designing primers for methylation PCRs. Bioinformatics.

[33] Wright S (1922). Coefficients of Inbreeding and Relationship. Am Nat.

