# Supplementary material for: Putative regulation mechanism for the MSTN gene by a CpG island generated by the SINE marker Ins227bp
Source: BMC Vet Res. 2015 Jun 23;11:138. doi: 10.1186/s12917-015-0428-3 (PMC4476204; doi:10.1186/s12917-015-0428-3)
Supplement: Additional file 1: Figure S1. — Llinkage disequilibrium pairwise tested for g.66493737 T ≻ C and Ins227bp as well as for g.66493737 T ≻ C and BIEC2-417495. [file 12917_2015_428_MOESM1_ESM.pdf]

Linkage Disequilibrium

Haploview 4.2

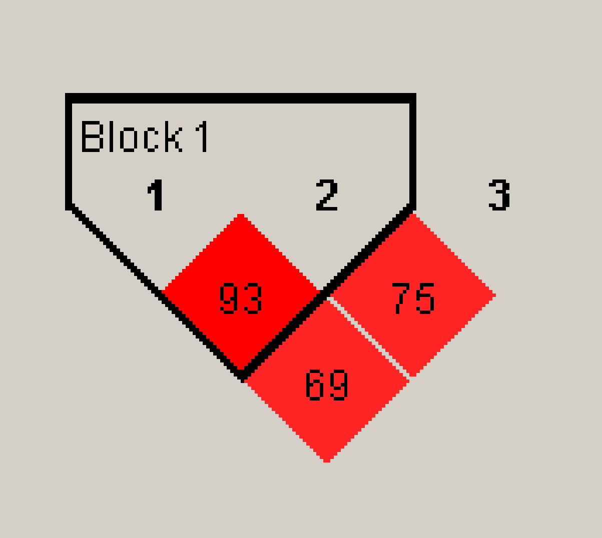

LD Plot

1: Speedgene polymorphism (intron 1 of *MSTN*)

2: INS227bp

3: BIEC2 (BIEC2-417495)

Haploview 4.2
29 April, 2008
http://www.broadinstitute.org/haploview/haploview

Citation: Barrett JC, Fry B, Maller J, Daly MJ.
Haploview: analysis and visualization of LD and haplotype maps.
Bioinformatics. 2005

Daly Lab at the Broad Institute
Cambridge, MA 02141, USA

Jeffrey Barrett
Julian B. Maller
David J. Bender
Jesse C. Whitworth
haploview@broad.mit.edu

Haploview can be cited with the following **paper**:

Barrett JC, Fry B, Maller J, Daly MJ. Haploview: analysis and visualization of LD and haplotype maps. Bioinformatics. 2005 Jan 15 [PubMed ID: 15297300]

Citations: 7340 (January 2015)

LD in the Hill article, BMC Genomics, 2010:

- LD was highest between g.66493737C>T and BIEC2-417495 (r^2 = 0.86).
- LD between g.66493737C>T and Ins227bp was r^2 = 0.73

SHEsis (http://analysis.bio-x.cn/myAnalysis.php)

r^2:	INS227bp	BIEC2
speedgene	0.931	0.699
INS227bp	-	0.758

D':	INS227bp	BIEC2
speedgene	1.000	0.915
INS227bp	-	0.919

Cite "Shi YY, He L. SHEsis, a powerful software platform for analyses of linkage disequilibrium, haplotype construction, and genetic association at polymorphism loci. Cell Res. 2005 Feb;15(2):97-8." if you used this platform for research study.

Citations: 774 (January 2015)
