# Supplementary material for: Putative regulation mechanism for the MSTN gene by a CpG island generated by the SINE marker Ins227bp
Source: BMC Vet Res. 2015 Jun 23;11:138. doi: 10.1186/s12917-015-0428-3 (PMC4476204; doi:10.1186/s12917-015-0428-3)
Supplement: Additional file 2: Table S1. — Details of genotyping assays. [file 12917_2015_428_MOESM2_ESM.pdf]

Supplemental information

Table S1 Details of genotyping assays

Polymorphism<br>(Reference)	Position on chromo-some 18	Assay type
Ins227bp<br>(Hill et al. 2010)	66495327 [Ins227bp] 66495326	qPCR
g.66493737C/T<br>(GenBank:<br>AY840554.2)	66493737	Dye-based ARMS-qPCR
BIEC2-417495<br>(Binns et al. 2010; Hill et al. 2010), BLAT)c	67186093	Probe-based ARMS-qPCR

Tabel S1 (cont)

Polymorphism	Sequence of oligonucleotide (5' to 3')	Tm (°C)[a]	Length (bp)	Amplicon length (bp)	Amplicon Tm (°C)[b]
Ins227bp	F: TGACAGCGAGATTCATTGTGG	58.3	21	174 and 401	77.8 and 83.9
	R: CTTTTGAGCAATGCCAAGCA	58.6	20		
g.66493737C/T	F: CTTTATCTACTTTAAACAGGACACAACA	55.6	28	78	76.4 to 77.3
	R(T): TGATAGCAGAGTCATAAAGGAAAATgAT	58.8	28		
	R(C): TGATAGCAGAGTCATAAAGGAAAATaAC	56.7	28		
BIEC2-417495	F(C): AATACCTTGATTGATTGGTTTTAcAC	55.7	26	160	not applicable
	F(T): AATACCTTGATTGATTGGTTTTAcAT	55.4	26		
	R: AGATGATGGTTAGACTTTGTGTCTGAT	57.5	27		
	Probe: FAM-CCTTCTTCAATCTCTGTTCCCTGGCCT-BHQ1	67.3	27		

Underlined and small letters in the sequence of a SNP-specific primer designate the targeted nucleotide and the artificial mismatch introduced to increase specificity, respectively.

F(C), F(T), R(C), R(T): forward or reverse primer specific for C or T allele, respectively

Tm: melting temperature

[a]predicted by Primer Express 2.0 software (Life Technologies); in case of SNP-specific primers, Tm was predicted for the

completely matching sequence

[b]determined by dissociating products of fluorogenic dye-based qPCR

[c]amplicon spans positions 67,186,068 to 67,186,227 in the alignment tool BLAT (BLAST-like Alignment Tool)
